# Supplementing long-chain *n*-3 polyunsaturated fatty acids in canned wild Pacific pink salmon with Alaska salmon oil

**DOI:** 10.1002/fsn3.4

**Published:** 2013-01-08

**Authors:** Trina J Lapis, Alexandra C M Oliveira, Charles A Crapo, Brian Himelbloom, Peter J Bechtel, Kristy A Long

**Affiliations:** 1 Kodiak Seafood and Marine Science Center, School of Fisheries and Ocean Sciences, University of Alaska Fairbanks118 Trident Way, Kodiak, Alaska, 99615-7401; 2 USDA-ARS Subarctic Agricultural Research Unit, University of Alaska Fairbanks118 Trident Way, Kodiak, Alaska, 99615-7401; 3 Cooperative Extension Service, University of Alaska Fairbanks213 Cooperative Extension Building, Fairbanks, Alaska, 99775-6180

**Keywords:** Canned salmon, Pacific salmon, salmon oil, seafood composition

## Abstract

Establishing *n*-3 polyunsaturated fatty acid contents in canned wild Alaska pink salmon products is challenging due to ample natural variation found in lipid content of pink salmon muscle. This study investigated the effect of adding salmon oil (SO) to canned pink salmon produced from fish exhibiting two opposite degrees of skin watermarking, bright (B) and dark (D). Specific goals of the study were to evaluate the benefits of adding SO to canned pink salmon with regard to nutritional value of the product, sensory characteristics, and the oxidative and hydrolytic stability of the lipids over thermal processing. Six groups of canned pink salmon were produced with variable levels of SO, either using bright (with 0, 1, or 2% SO) or dark (with 0, 2, or 4% SO) pink salmon. Compositional analysis revealed highest (*P *<* *0.05) lipid content in sample B2 (8.7%) and lowest (*P *<* *0.05) lipid content in sample D0 (3.5%). Lipid content of samples B0, B1, D2, and D4 was not significantly different (*P *>* *0.05) ranging from 5.7% to 6.8%. Consequently, addition of SO to canned pink salmon allowed for consistent lipid content between bright and dark fish. Addition of 1% or 2% SO to canned bright pink salmon was not detrimental to the sensory properties of the product. It is recommended that canned bright pink salmon be supplemented with at least 1% SO, while supplementation with 2% SO would guarantee a minimum quantity of 1.9 g of *n*-3 fatty acids per 100 g of product. Addition of 4% SO to canned dark pink salmon was detrimental to product texture and taste, while supplementation with 2% SO did not negatively affect sensorial properties of the product. Accordingly, canned dark pink salmon should be supplemented with 2% SO so that a minimum *n*-3 fatty acids content of 1.5 g per 100 g of product.

## Introduction

In Alaska, pink salmon annual harvests averaged 175,000 t from 2005 to 2009, comprising about half of the total salmon catch (Alaska Department of Fish and Game 2009). Approximately, 55% of the pink salmon catch volume is processed into cans (Franz 2006). Between 2000 and 2004, U.S. consumption of Pacific salmon species averaged 284,000 tons, 16% of which was canned salmon (Knapp et al. 2007). Canned salmon is a staple of U.S. diet (Liese et al. 2007) with domestic consumption upwards of 60,000 tons on years of high catch volumes (Knapp et al. 2007). Canned salmon is also exported to Europe and Asia and is a staple food item in the United Kingdom and Japan (Knapp et al. 2007).

During the past decade, consumer awareness regarding the need to increase dietary intake of long chain *n*-3 polyunsaturated fatty acids (LC *n*-3 PUFA) has increased significantly (Ruxton et al. 2005). Research has shown that daily consumption of fatty fish or fish oil containing these fatty acids reduces risk of coronary disease and decrease progression of atherosclerosis in coronary patients (Simopoulos 1991, Harris et al. 2008). Balk et al. (2006) indicated that evidence supports a dose-dependent effect of fish oil on reducing levels of serum triglycerides and cholesterol. These fatty acids may also benefit treatment and prevention of systemic inflammatory diseases (Wall et al. 2010), such as ulcerative colitis, psoriasis (Simopoulos 1991), and rheumatoid arthritis (Calder 2006). Additional studies suggest that LC *n*-3 PUFA may reduce risk of progression of psychotic disorders, such as schizophrenia, in young people with subthreshold psychotic states (Amminger et al. 2010). Richardson (2006) reported on the potential effects of LC *n*-3 PUFA on patients displaying symptoms of attention deficit/hyperactivity disorder, while SanGiovanni and Chew (2005) described the role of LC *n*-3 PUFA in health and disease of the retina. Despite the many health benefits attributed to LC *n*-3 PUFA, controversy exists on their potential effects on reducing cancer risk (Larsson et al. 2004; MacLean et al. 2006). Larsson et al. (2004) described several mechanisms of action of LC *n*-3 PUFA in modifying the carcinogenic process. MacLean et al. (2006) conducted a systematic study that synthesized published and unpublished evidence in this area and concluded that no significant associations between LC *n*-3 PUFA consumption and cancer incidence were found for numerous types of cancers.

Pink salmon is high in protein and a good source of LC *n*-3 PUFA (˜30%), especially of 20:5*n*-3 (eicosapentaenoic acid, EPA) and 22:6*n*-3 (docosahexaenoic acid, DHA) (Kong et al. 2008). Nonetheless, there is high natural variability in the lipid content of wild Alaska pink salmon, which vary from 2% up to 9% (Hardy and King 1989). High natural variability in the lipid content of wild salmon makes the disclosure of LC *n*-3 PUFA content, and in particular of EPA and DHA contents, in the product nutritional label difficult. The main cause of variability of lipid content in wild Pacific salmon is sexual maturity and spawning migration (Ando et al. 1985). During spawning migration, the feeding activity of Pacific salmon decreases and stored lipids are used as energy, and this causes a decrease in total lipid content of muscle as fish near spawning (Ando et al. 1985; Durance and Collins 1991; Reid et al. 1993). Concomitantly, the lipid-soluble pigments responsible for the natural rose to orange color of salmon fillets migrate from flesh to skin, and in the case of females to the eggs (Durance and Collins 1991; Reid et al. 1993). Migration of pigments from flesh to skin cause a skin blushing effect, ordinarily referred to as “skin watermarking” (Ando et al. 1985; Huynh and Mackey 1990; Durance and Collins 1991). Alaska seafood processors use degree of skin watermarking as one of the grading parameters for Pacific salmon species, separating fish within each species as bright, semi-bright, and dark (Oliveira et al. 2005). Dark fish, most prevalent in the commercial catch during the late part of the salmon run, are also designated as pale-meat salmon because of the absence of the naturally occurring pigments in the flesh of heavily skin-watermarked fish. Huynh and Mackey (1990) conducted a quality study of late-run chum salmon and noted that muscle quality is greatly impacted by the biochemical changes that occur during spawning migration. Late-run salmon muscle often has less desirable texture and flavor, flesh softness, poor taste, and develops a distinct “late-odor” that reduces value of the product (Huynh and Mackey 1990). When compared with canned pink salmon produced from bright grade A fish, pale-meat canned pink salmon had a distinct profile of chemical volatiles; notwithstanding, a specific chemical compound that imparted “late-odor” notes to product was not readily identifiable (Oliveira et al. 2005).

In the early days of the Alaska salmon industry, canning often included the addition of salmon oil (SO) rendered from salmon heads. However, in the late 1960s, most canned salmon processors in Alaska discontinued this practice, and production of SO for human use ceased. In the last decade, growing interest in fish oils due to their nutritional benefit has increased the price of this commodity, prompting a revival of edible SO production in Alaska (Bimbo 2009). Pacific salmon stores lipids in their head and content of oil, despite variable between species, may be as high as 16% w/w (Sathivel et al. 2005; Smiley et al. 2010). Salmon heads are a major byproduct of salmon processing, and currently the production of edible SO in Alaska is a lucrative business. SO produced in Alaska contains about 65–93 mg/g oil EPA and 74–102 mg/g oil DHA (Oliveira et al. 2010), and is the only optional ingredient other than salt permitted by the standard of identity for canned Pacific salmon by the Code of Federal Regulation (21 CFR 161.70) (United States Food and Drug Administration 2010). Adding SO to canned Alaska pink salmon will boost the lipid content of heavily watermarked fish, or pale-meat pink salmon, improving its nutritional value and product consistency. This study investigated the effect of adding Alaska edible wild SO to canned Alaska pink salmon produced from fish exhibiting two opposite degrees of skin watermarking, bright and dark. Specific goals of the study were to evaluate the benefits of adding SO to canned pink salmon with regard to nutritional value of the product, sensorial characteristics, and the oxidative stability of the lipids over thermal processing.

## Materials and Methods

### Fish procurement and processing

A total of 250 pink salmon (*Oncorhynchus gorbuscha*) were procured from a processing plant in Kodiak, Alaska. Fish were seine-caught near Afognak Island, Kodiak Archipelago, Alaska, during the summer of 2008. Of which, 125 pink salmon exhibited no signs of skin watermarking and were commercially graded as grade A bright fish, while the remaining 125 pink salmon procured were heavily skin-watermarked and received a commercial graded of dark. Fish were less than 24 h postmortem and gutted using an iron butcher at the commercial plant. Fish were brought in iced totes to the Kodiak Seafood and Marine Science (University of Alaska, Kodiak, AK) pilot plant and canned the same day. A quantity of 15 L of human-grade SO, rendered from salmon heads during summer of 2008, was procured from a SO processor (Alaska Protein Recovery, Juneau, AK; http://alaskaproteinrecovery.com/salmonoil) in August of 2008.

Bright pink salmon were cut into 215 g steaks and placed in 307 × 200.25 cans with 3 g NaCl and 0, 1, or 2% (w/w) human-grade Alaska SO, and mirrored standard commercial salmon canning practices in Alaska. Dark pink salmon were processed in identical fashion; however, the levels of SO added to cans were 0, 2, or 4% (w/w). The cans were filled at room temperature. The total weight of SO added was converted to the corresponding volume using product density, 0.9 g/mL (Sathivel 2005), and values were rounded off such that 1, 2, and 4% corresponded to 2.5, 5, and 10 mL, respectively. The two-piece cans were vacuum-sealed, retorted at 120°C for 69 min and water-cooled (National Food Processors Association 1982). A total of 960 cans of pink salmon, 160 for each of the six treatments, were produced. The treatments were canned bright pink salmon with 0% (B0), 1% (B1), and 2% (B2) human-grade SO, and canned dark pink salmon with 0% (D0), 2% (D2), and 4% (D4) human-grade SO.

### Canned salmon sampling for chemical analyses

After 8–10 months of storage at room temperature (20–24°C), 24 canned pink salmon samples were randomly selected from each of the six groups (B0, B1, B2, D0, D2, and D4) for chemical analyses. Two cans within a treatment (including bone, skin, and liquid) were homogenized at a time, using a Waring Commercial laboratory blender (Blender 7012S, Torrington, CT), to produce one sample that contained approximately 450 g of product, which was sufficient material to conduct all chemical analyses planned. Therefore, 12 sample replicates were produced from each of the six treatments, each composed of contents from two identical salmon cans.

Samples were individually frozen to −30°C overnight in a tray placed in a walk-in freezer (Bally®, Morehead City, NC). Frozen samples were placed in the freeze drier (VirTis Virtual 52ES Freeze Dryer Lyophilizer, Gardiner, NY) and maintained at −30°C for 30 min then at −40°C for 30 min with a condenser temperature of −50°C and chamber pressure of 53.33 kPa. The primary freeze drying parameters for shelf temperature and drying time were −40°C for 6 h, −30°C for 5 h, −20°C for 4 h, −10°C for 3 h, and 0°C for 2 h, all under 8 Pa. The secondary drying was set at 25°C for 3 h at 8 Pa. The freeze-drying process took 24 h and was based on processing parameters established for pink salmon fillets (Crapo et al. 2010). The freeze-dried samples removed from the freeze-drier chamber and immediately comminuted to powder using a Mr. Coffee® IDS-50 coffee grinder (Shelton, CT). A quantity of 0.5 g of sample from each tray was used to measure water activity (a_W_), which was determined using an AquaLab® water activity meter (Series 3 TE, Pullman, WA). Upon verification that all samples had water activity equal or below 0.2, samples were vacuum packaged (Koch Ultravac® 2100, Kansas City, MO) and promptly frozen at −30°C until chemically analyzed.

### Proximate composition analysis

A total of 12 samples from each sample group (B0, B1, B2, D0, D2, and D4), each containing the entire contents of two identical cans of pink salmon, were used for analysis. Moisture content was the only parameter measured for wet samples, while moisture, ash, protein, and lipid contents were determined using the freeze-dried samples counterparts. Moisture and ash contents were determined using Official Methods of Analysis of AOAC International (AOAC) methods #952.08 and #938.08, respectively (AOAC 2005). Nitrogen content was accessed by pyrolysis, as described in AOAC method 968.06 with a LECO FP-2000 nitrogen analyzer (LECO Co., St. Joseph, MO), and protein content was calculated as 6.25 times % N (AOAC 2005). Lipids were determined gravimetrically using an ASE200 Accelerated Solvent Extractor (Dionex, Sunnyvale, CA) using an adaptation to the procedure previously described by Oliveira et al. (2006). Approximately, 4–5 g of freeze-dried samples were mixed with an equal amount of hydromatrix (Varian, Inc., Palo Alto, CA), and accurate sample weights were recorded using an analytical balance (AX105 DeltaRange**®**, Mettler Toledo, Columbus, OH). The mixture was placed in a 33 mL extraction cell with a cellulose filter and quartz sand (Accusand®, Unimin Corp., Le Sueur, MN) to fill dead-volume at both ends of the cell. Lipids were extracted using dichloromethane as solvent, and the extraction parameters were 1500 psi pressure, 100°C, and three static cycles of 5 min extraction for each sample producing a total of 50–55 mL extracted volume. For the lipid yield data, the solvent extract collected in a preweighed 60 mL collection vial was dried at 40°C under a stream of nitrogen until constant weight using a Turbovap LV (Caliper Life Sciences, Hopkinton, MA). Extracted lipids were dissolved in hexane with 0.01% butylated hydroxytoluene (BHT), at a ratio of about 1:10, and kept at −80°C until further analysis. Proximate composition data, reported on a wet weight basis, was calculated using moisture content of wet samples, and lipid, protein and ash contents determined in their freeze-dried counterparts. The proximate composition values on a wet weight basis were also adjusted using the moisture contents of each of the freeze-dried samples to the moisture level of the corresponding sample determined from wet tissues.

### Salt content analysis

A total of 12 freeze-dried samples from each sample group (B0, B1, B2, D0, D2, and D4), each containing the entire contents of two identical cans of pink salmon, were used for analysis. One gram of freeze-dried sample was weighed into 9-mL screw-capped test tube and 8 mL of deionized water added. The contents were mixed for 1 min using a mini vortexer (VWR, West Chester, PA) then centrifuged at 2000 rpm using a Centra CL2 Thermo IEC benchtop centrifuge (Thermo Fisher Scientific, Inc., Waltham, MA) for 30 min. The supernatant liquid was isolated and diluted 1:15 with deionized water. The resulting mixture was analyzed for salt content using an M926 Chloride Analyzer (Nelson-Jameson, Inc., Marshfield, WI) and reported as mg/100 g of wet sample.

### Fatty acids analysis

Fatty acid methyl esters were prepared, in duplicates, using 20 mg of lipids extracted with the ASE200 from each freeze-dried sample, which had been extracted. Fatty acid methyl esters were also prepared, in duplicates, using 20 mg of SO (Alaska Protein Recovery, Juneau, AK; http://alaskaproteinrecovery.com/salmonoil). The esterification procedure followed the method described by Maxwell and Marmer (1983) using 1 mg of tricosanoic acid methyl ester as internal standard. Fatty acid methyl esters were transferred into 1.5-mL snap-cap amber vials (Agilent Technologies, Wilmington, DE) and immediately analyzed using a gas chromatographer (GC) model 6850 (Agilent Technologies, Wilmington, DE) fitted with a DB-23 (60 m × 0.25 mm id., 0.25 μm film) capillary column (Agilent Technologies, Wilmington, DE). Hydrogen was used as the carrier gas at a constant flow of 1.0 mL/min and average velocity of 30 cm/sec. The initial nominal inlet pressure was 15.26 psi, total flow was 58.6 mL/min, and temperature was 250°C. The inlet was operated in split mode at 50:1 ratio, and the oven programming was as follows: 140–180°C at 2°C/min, 180–200°C at 2.5°C/min, 200–210°C at 0.5°C/min, and 210–230°C at 10°C/min. Total analysis time was 50 min. The GC was coupled to a flame ionization detector operated at 275°C. Detector make-up gas flow was 35 mL N_2_/min, and air and hydrogen flows were 450 mL/min and 40 mL/min, respectively. An auto-sampler performed the GC injections of standards and samples, and injection volume was 1 μL. Data were collected and analyzed using the GC ChemStation program (Rev.A.08.03 [847], Agilent Technologies 1990-2000, Wilmington, DE). Identification of peaks was performed using the following Supelco® (Bellefonte, PA) standards: Marine Oil #1, Marine Oil #3, S189-19, and Bacterial Acid Methyl Esters Mix. Results were determined as milligrams of fatty acids per gram of oil then converted to mg/100 g of product (serving size stipulated in this study) based on the fat content of the sample.

### Lipid hydrolysis and oxidation analysis

Lipid hydrolysis and lipid oxidation parameters were determined, in duplicates, for canned pink salmon samples and for the SO (Alaska Protein Recovery, Juneau, AK; http://alaskaproteinrecovery.com/salmonoil) using American Oil Chemists' Society methods (AOCS 2004). The free fatty acid values (FFA, reported as % oleic acid), peroxide values (PV), and 2-thiobarbituric acid (TBA) followed AOCS methods # Ca 5a-40, Cd 8-53, and Cd 19-90, respectively (AOCS 2004).

### Consumer attribute analysis test

A group of eight people (faculty and graduate students) from the University of Alaska Seafood Science Graduate Program (KSMSC, Kodiak, AK) defined the attributes used during the Consumer Test during a preliminary sensory evaluation of the canned pink salmon samples. The group tasted and smelled samples from each of the six treatments (B0, B1, B2, D0, D2, and D4) and determined attributes most relevant in the samples to be appearance, color, overall taste, saltiness, bitterness, fattiness, fish flavor, and texture. The main sensory evaluation conducted was a consumer attribute analysis (CAA) test as previously described by Oliveira et al. (2004). The CAA test was conducted in October of 2008 at the University of Alaska Fairbanks (UAF) campus, Fairbanks (Alaska), with the assistance of the Cooperative Extension Service (CES) personnel of UAF. The panelists were community members of Fairbanks, and students, staff, and faculty of UAF ranging in age from 18 to 80 years old. The age average of participants was 31 years old, the ratio of females to males was approximately 1:1.5 and about 8% of the participants did not disclose gender information. Panelists were selected based on their liking of fish and fishery products and their frequency of eating fish, which should be at least twice a month.

Unstructured scales (15 cm) with verbal anchors on both ends were used (Oliveira et al. 2004), and instruction on the use the scale was given to each panelist. Each can of sample was opened and the liquid, commercially designated as canned salmon liquor, was poured equally into four plastic cups with lids (118 mL volume). Canned pink salmon steak was divided into four equal parts and placed into the cups with their respective portioned liquor. Unsalted crackers and commercial bottled water were provided for palate rinsing. The panelists were asked to evaluate the samples from left to right. Panelists rated samples based on the sensory attributes requested by placing a vertical line anywhere within the scale where they thought best described the sample. The panelists' scores were determined by measuring the length (cm) with a ruler from the left verbal anchor to the vertical line placed by the panelist for each sample code. Scores were recorded up to 1 decimal place with 0 and 15 as lower and upper limits, respectively, for all attributes. The CAA Test was conducted through four consecutive days, and in day 1 and day 2, product comparisons carried out were B0 versus B1 versus B2 (107 panelists) and D0 versus D1 versus D2 (103 panelists), respectively. In day 1 and 2, each participant received one tray containing three sample cups. In day 3, two separate product comparisons were conducted, B0 versus D0 (101 panelists) and B1 versus D2 (105 panelists), and each panelist received two separate trays each containing two sample cups. Order of presentation for first and second sample pairs was randomized. Similarly, in day 4, two separate product comparisons were conducted, B2 versus D4 (103 panelists) and B2 versus D4 (105 panelists). In day 1 and 2 of the CAA test, approximately 90 cans of salmon were used each day, while in days 3 and 4 about 120 cans were used each day. In total, the CAA test required approximately 420 cans of salmon.

### Statistical analysis

Significant differences between canned salmon groups were determined using one-way analysis of variance (ANOVA; *P* < 0.05) followed by Tukey's Honestly Significance Difference Test (*P* < 0.05). All analyses were conducted using Statistica version 8.0 (StatSoft Inc., Tulsa, OK). Results for chemical analysis are reported as weighted means (*n* = 12) and respective standard deviations for each canned salmon group (B0, B1, B2, D0, D2, and D4). Results for sensory analysis are reported as the average of sensory scores and respective standard deviations.

## Results and Discussion

### Proximate composition

The a_W_ values were below 0.2 for all freeze-dried samples. The average moisture contents of freeze-dried samples were 3.0% for B0, 3.0% for B1, 2.3% for B2, 2.6% for D0, 2.7% for D2, and 4.1% for D4. For canned bright and dark Alaska pink salmon with different levels of SO, protein content was significantly lower (*P* < 0.05) in D4 and highest in B0, while ash content was lowest in B0 and highest in D0, D2, and D4 (Table 1). The principal variations were observed in the moisture and lipid contents, which were inversely related. Moisture content was significantly higher (*P* < 0.05) in D0 and lowest in B2, while lipid content was significantly higher (*P* < 0.05) in B2 and lowest in D0 (Table 1). The addition of 4% SO (D4) was expected to have the highest lipid content or at least equivalent to B2. Due to the variation in the intrinsic qualities between individual fish from the same run, the lipid contents of B0, B1, D2, and D4 were not significantly different (*P* > 0.05). This finding supports observation that pink salmon show a decrease in lipid content as they stop feeding during spawning (Kitahara 1983; Ando et al. 1985; Reid et al. 1993). The lipid content of canned dark pink salmon with added SO (D2 and D4) showed no significant difference (*P* > 0.05) to canned bright pink salmon without added SO (B0). More importantly, this implied that 2% or 4% SO added prior to commercial canning of dark fish would result in consistent lipid content in the product.

**Table 1:** Proximate composition (% w/w ± SD) of canned Alaska pink salmon with different levels of salmon oil

	B0 (*n *=* *12)	B1 (*n *=* *12)	B2 (*n *=* *12)	D0 (*n *=* *12)	D2 (*n *=* *12)	D4 (*n *=* *12)
Moisture	71.0^c^ ± 0.4	70.7^c^ ± 0.7	69.0^d^ ± 1.2	74.4^a^ ± 0.2	72.5^b^ ± 0.6	72.2^b^ ± 0.4
Protein	20.5^a^ ± 0.3	20.1^ab^ ± 0.2	19.6^bc^ ± 0.2	19.2^c^ ± 0.1	18.9^cd^ ± 0.1	18.1^d^ ± 0.1
Lipid	5.8^b^ ± 1.2	6.5^b^ ± 1.6	8.7^a^ ± 2.4	3.5^c^ ± 0.6	5.7^b^ ± 0.6	6.8^b^ ± 0.6
Ash	2.6^b^ ± 0.8	2.7^ab^ ± 0.9	2.7^ab^ ± 1.0	2.9^a^ ± 0.4	2.9^a^ ± 0.4	2.9^a^ ± 0.5
Salt	1.36^c^ ± 0.11	1.38^bc^ ± 0.22	1.46^abc^ ± 0.22	1.57^ab^ ± 0.09	1.63^a^ ± 0.12	1.48^abc^ ± 0.16

Treatments are canned bright pink salmon with 0% (B0), 1% (B1), and 2% (B2) human-grade salmon oil, and canned dark pink salmon with 0% (D0), 2% (D2), and 4% (D4) human-grade salmon oil. Different superscript letters within a row indicate significant differences (*P* < 0.05); SD standard deviation of the mean.

Shostrom et al. (1924), studying traditional canned pink salmon (bone-free) obtained from nine different districts in Alaska at the end of the canning season, suggested a wide variation in lipid content (4–8%). Similarly, 5–13% lipid content was determined in traditional cans produced during early and late pink salmon runs from the north and south coasts of British Columbia (Vanderstoep et al. 1990). Also, traditional canned pink salmon produced by three different processors in Japan, in October 1987 and June and November 1988, had 4–15% lipid content (Sasaki et al. 1989). These reports imply a variation in the sexual maturity of the pink salmon sample and the lipid contents of B0 and D0 in the present study confirmed the hypothesis. Furthermore, the lipid contents (4.7% and 5.3%) for two commercial samples of traditional canned pink salmon purchased from Lafayette, IN stores (Shim et al. 2004) were within the range observed for B0 and D0. The USDA (2012) data for canned pink salmon, solids with bone and liquid, were 74.04% moisture, 4.97% lipid, 19.68% protein, and 1.31% ash and were close to the values given in Table 1.

Aside from pink salmon, other species of salmon are canned. Coho salmon (*O. kisutch*), following a traditional canning method used in villages in interior Alaska, showed a low 2% lipid content and high 6% ash content (Bower et al. 2007). In their study, spawning coho salmon were harvested and brined before canning, which allowed uptake of moisture in fillets and making lipid content proportionally lower. Farmed coho salmon (La Coruña, Spain) canned in sunflower oil resulted in higher lipid content (3–4%) than unsupplemented product (Rodriguez et al. 2009). Sockeye salmon (*O. nerka*) from two different processors in Japan contain 7–8% lipid in the cans (Ota et al. 1990). Other commercially canned fish species contain lipids ranging from 6% to 8% for light tuna, 5% to 6% for white and albacore tuna, and 4% to 5% for mackerel (Shim et al. 2004).

### Salt content

The salt content in pink salmon cans ranged from 1.36% to 1.63% of the total wet weight of the cans (Table 1). There were significant differences detected, and D2 had the highest salt content while B0 had the lowest (*P* < 0.05). Overall, the 0.27% salt content difference between highest and lowest values is relatively small, and significant differences reflect precision of the measurement. The range in salt content observed in this study is slightly higher than the 0.8–1.4% for commercial British Columbia canned pink salmon (Vanderstoep et al. 1990). In their study, 1.8–2 g of salt were added per 213 g can of salmon compared with the 3 g of salt per 215 g can of salmon in this study, which followed Alaska salmon canning industry practices.

### Fatty acid profiles

Table 2 shows the fatty acid profile for SO, which was typical of commercial Alaska SO (Oliveira et al. 2010). The fatty acid profiles of canned bright and dark pink salmon are also presented in Table 2. Among the saturated fatty acids (SAT), palmitic acid (16:0) was the most abundant, with B2 having the significantly highest (*P* < 0.05) value and D0 the lowest. The palmitic acid content of samples B0, B1, D2, and D4 was not significantly different from each other. The most abundant monounsaturated fatty acid (MUFA) found in all samples was oleic acid (18:1*n*-9 *cis*), with B2 having the highest concentration and D0 the lowest. Cetoleic acid (22:1*n*-11) and gadoleic acid (20:1*n*-11) were second and third, respectively, in abundance among the MUFAs in all samples. These two fatty acids are exogenous in origin and the concentrations in salmon reflect feeding activity (Ackman 1999). Cetoleic and gadoleic acids were significantly lowest (*P* < 0.05) in D0 indicating a decrease in feeding activity of pink salmon during spawning. The two most abundant LC *n*-3 PUFA in the canned pink salmon were DHA and EPA. The DHA content was most abundant in B2 and least abundant in D0 (*P* < 0.05), while not significantly different (*P* > 0.05) among B0, B1, D2, and D4. The control and supplemented samples from this study had lower DHA content than the 1300–1400 mg/100 g for canned pink salmon (drained meat) from British Columbia (Ackman 1996). The DHA content of B0, B1, B2, and D2 is in the range reported for commercially canned pink salmon (564–874 mg DHA/100 g of product) purchased in Indiana (Shim et al. 2004), and for canned salmon purchased in Japan which contained 575–906 mg DHA/100 g of product (Sasaki et al. 1989). The EPA content was significantly highest (*P* < 0.05) in B2 and lowest in D0 but all samples were lower as compared with previous reports on canned pink salmon. The British Columbia product contains 700–900 mg EPA/100 g of product (Ackman 1996), while two of the three purchased products from Japan contained 1200–1300 mg EPA/100 g of product (Sasaki et al. 1989) and was close to those for B0, B1, D0, D2, and D4. The DHA:EPA showed D0 having the significantly highest (*P* < 0.05) value while B2 and D4 shared the lowest ratio. Some studies suggest that DHA may be more cardio-protective than EPA and being more effective in lowering postprandial triglyceride levels (Grimsgaard et al. 1997; Hansen et al. 1998), slowing the resting pulse rate (Grimsgaard et al. 1998), and decreasing blood pressure (Bao et al. 1998). British Columbia canned pink salmon (drained meat) had 1.7–1.8 DHA:EPA (Ackman 1996), which was similar to B0, B1, and D2. Lower DHA to EPA ratios (0.6–1) were recorded for canned pink salmon purchased in Indiana (Shim et al. 2004), and the range of 0.6–0.8 for two of three types of canned pink salmon purchased in Japan (Sasaki et al. 1989). A third sample of canned pink salmon purchased in Japan had a DHA:EPA of 2 (Sasaki et al. 1989), which was similar to that for D0. Compared with other species of canned salmon, the DHA to EPA ratios of 1.5–2 for canned sockeye salmon from Japan (Ota et al. 1990) are similar with that of pink salmon from this study. On the other hand, canned coho salmon from interior Alaska showed a higher DHA:EPA of 2.4 (Bower et al. 2007). The majority of commercial canned light tuna and albacore tuna purchased in Indiana has a higher DHA to EPA ratio ranging from 3 to 8 (Shim et al. 2004). However, the EPA and DHA contents for canned tuna were lower than those determined from this study and were 32–58 mg/100 g of product and 181–300 mg/100 g of product, respectively, except for one sample of canned albacore tuna with 190 mg EPA/100 g of product and 741 mg DHA/100 g of product (Shim et al. 2004). Canned mackerel purchased in Indiana has a lower DHA:EPA of 1.2–1.3 (Shim et al. 2004) than that of the canned pink salmon in this study.

**Table 2:** Fatty acid profiles of salmon oil (mg/g oil) and canned bright and dark pink salmon with different levels of salmon oil (mg/100 g of products** ± **SD)

	SO (*n *=* *1)	B0 (*n *=* *12)	B1 (*n *=* *12)	B2 (*n *=* *12)	D0 (*n *=* *12)	D2 (*n *=* *12)	D4 (*n *=* *12)
14:0	44.92	203^b^ ± 45	203^b^ ± 68	296^a^ ± 85	85^c^ ± 24	209^b^ ± 26	213^b^ ± 43
16:0	117.18	589^b^ ± 102	581^b^ ± 189	856^a^ ± 191	297^c^ ± 87	612^b^ ± 62	625^b^ ± 122
16:1n-7	37.33	141^cd^ ± 30	136^d^ ± 40	230^a^ ± 50	78^e^ ± 25	191^ab^ ± 24	181^bc^ ± 37
18:0	24.15	103^b^ ± 12	105^b^ ± 26	153^a^ ± 33	54^c^ ± 18	121^b^ ± 12	122^b^ ± 24
18:1*n*-9 *trans*	6.88	53^ab^ ± 13	51^ab^ ± 16	67^a^ ± 22	22^c^ ± 8	51^ab^ ± 6	43^b^ ± 9
18:1*n*-9 *cis*	120.08	436^c^ ± 77	477^bc^ ± 142	729^a^ ± 150	247^d^ ± 84	579^b^ ± 65	589^b^ ± 115
18:1*n*-7	18.33	89^bc^ ± 20	95^b^ ± 41	132^a^ ± 21	57^c^ ± 19	108^ab^ ± 18	108^ab^ ± 21
18:2*n*-6 *trans*	5.48	35^b^ ± 8	33^b^ ± 11	53^a^ ± 15	15^c^ ± 4	37^b^ ± 5	33^b^ ± 8
18:2*n*-6 *cis*	13.40	71^b^ ± 12	67^b^ ± 21	101^a^ ± 28	34^c^ ± 12	77^b^ ± 9	73^b^ ± 15
18:3*n*-3	12.35	55^b^ ± 10	55^b^ ± 17	84^a^ ± 24	26^c^ ± 9	66^b^ ± 7	63^b^ ± 13
18:4*n*-3	23.20	103^bc^ ± 41	103^bc^ ± 35	166^a^ ± 53	59^c^ ± 22	137^ab^ ± 23	122^b^ ± 28
20:1*n*-11	42.52	392^ab^ ± 130	413^ab^ ± 157	509^a^ ± 234	144^c^ ± 49	301^bc^ ± 52	303^bc^ ± 73
20:1*n*-9	21.46	137^b^ ± 36	141^b^ ± 45	187^a^ ± 60	62^c^ ± 22	130^b^ ± 21	123^b^ ± 24
20:4*n*-3	15.67	68^b^ ± 14	71^b^ ± 21	105^a^ ± 29	32^c^ ± 11	81^b^ ± 10	78^b^ ± 16
20:5*n*-3 (EPA)	92.86	344^b^ ± 81	332^b^ ± 110	534^a^ ± 112	179^c^ ± 54	394^b^ ± 42	419^b^ ± 85
22:1*n*-11	57.98	424^ab^ ± 116	431^ab^ ± 146	571^a^ ± 244	181^c^ ± 73	396^b^ ± 75	353^bc^ ± 76
22:1*n*-9	13.33	34^b^ ± 10	38^b^ ± 11	60^a^ ± 16	18^c^ ± 7	53^a^ ± 8	54^a^ ± 10
22:5*n*-3	24.23	91^d^ ± 12	93^cd^ ± 24	139^a^ ± 22	49^e^ ± 17	116^ab^ ± 13	115^bc^ ± 23
22:6*n*-3 (DHA)	99.35	598^b^ ± 66	583^b^ ± 133	851^a^ ± 212	354^c^ ± 117	649^b^ ± 47	641^b^ ± 124
24:1*n*-9	6.14	40^b^ ± 9	40^b^ ± 12	58^a^ ± 19	20^c^ ± 6	43^b^ ± 5	39^b^ ± 9
DHA/EPA	1.1	1.8^ab^ ± 0.4	1.8^ab^ ± 0.3	1.6^b^ ± 0.1	2.0^a^ ± 0.2	1.7^ab^ ± 0.1	1.5^b^ ± 0.1

Treatments are canned bright pink salmon with 0% (B0), 1% (B1), and 2% (B2) human-grade salmon oil, and canned dark pink salmon with 0% (D0), 2% (D2), and 4% (D4) human-grade salmon oil. Values of less than 35 mg/100 g of product were not reported as individual fatty acids. Different superscript letters within a row indicate significant differences (*P* < 0.05). SD, standard deviation of the mean; EPA, eicosapentaenoic acid; DHA, docosahexaenoic acid.

The summary of fatty acid classes for SO and canned bright and dark pink salmon are shown in Table 3. The most abundant fatty acid class in all samples was MUFA, which was highest in B2 (*P* < 0.05), lowest in D0 (*P* < 0.05), and not significantly different (*P* > 0.05) between B0, B1, D2, and D4. Least abundant among fatty acid classes was SAT, ranging from 0.5 to 1.4 g/100 g of product. This range was low considering that the American Heart Association (AHA 2006) recommendation is <16 g saturated fat intake per day (<7% of energy) for cardiovascular disease risk reduction in the general population. Canned pink salmon can be an important component of a healthy diet to reduce risk of death from coronary arterial disease (AHA 2006). Conversely, *n*-3 fatty acids contents of the samples were high having an abundance of 1.9 g/100 g of product in B2 and 0.7 g/100 g of product in D0, the difference of which was significant (*P* < 0.05). Simopoulos et al. (1999) recommend an Adequate Intake (AI) of EPA+DHA for adults of 650 mg/day. Applied to this study, 100 g of product of each of the canned samples could provide the AI for EPA+DHA except for D0, which gave the lowest EPA+DHA value. Sample B2 had a significantly higher (*P* < 0.05) EPA+DHA value, while samples B0, B1, D2, and D4 were not significantly different. In addition, AHA recommends consumption of 1 g of EPA+DHA per day for patients with documented coronary heart disease (AHA 2006). Consumption of 100 g per day of B2, as well as samples B0, B1, D2, and D4 can provide adequate EPA+DHA requirement for patients with documented coronary heart disease. In summary, conventionally canned bright pink salmon (B0) may provide the AI for EPA+DHA for adults and AHA's recommendation for daily intake for patients with coronary heart disease, while canned unsupplemented dark fish (D0) may not. The significant difference between the EPA+DHA values of B0 and D0 reflects the natural variation, thus inconsistent lipid-related composition and nutritional quality of canned pink salmon. This variation can become a concern if consumption of canned pink salmon is recommended as part of coronary heart disease patients' diets. In this study, the concern was resolved with the addition of SO to canned dark pink salmon. The EPA+DHA values for traditional canned pink salmon determined from other studies were higher: 2.1–2.3 g/100 g of drained meat product from British Columbia (Ackman 1996), 0.9–2.1 g/100 g and 1.5–1.8 g/100 g of product from those purchased in Japan (Sasaki et al. 1989) and Indiana (Shim et al. 2004), respectively. In comparison, canned coho salmon had 0.5 g of EPA+DHA/100 g of product (Bower et al. 2007), while canned sockeye from Japan had 1.3–1.4 g/100 g of product (Ota et al. 1990). Other canned fish species contained 0.1–0.3 g/100 g of product for light tuna, 0.2–0.9 g/100 g of product for albacore tuna, and 0.5–1.2 g/100 g of product for mackerel (Shim et al. 2004).

**Table 3:** Summary of the fatty acid classes and contents of EPA and DHA in canned bright and dark pink salmon with different levels of salmon oil (g/100 g product ± SD)

	B0 (*n *=* *12)	B1 (*n *=* *12)	B2 (*n *=* *12)	D0 (*n *=* *12)	D2 (*n *=* *12)	D4 (*n *=* *12)
ΣSAT	1.0^b^ ± 0.2	1.0^b^ ± 0.3	1.4^a^ ± 0.3	0.5^c^ ± 0.1	1.1^b^ ± 0.1	1.1^b^ ± 0.2
ΣMUFA	1.9^b^ ± 0.3	1.9^b^ ± 0.6	2.7^a^ ± 0.8	0.9^c^ ± 0.3	2.0^b^ ± 0.2	1.9^b^ ± 0.4
ΣPUFA	1.4^b^ ± 0.2	1.4^b^ ± 0.4	2.1^a^ ± 0.5	0.8^c^ ± 0.2	1.6^b^ ± 0.1	1.6^b^ ± 0.3
EPA+DHA	0.9^b^ ± 0.1	0.9^b^ ± 0.2	1.4^a^ ± 0.3	0.5^c^ ± 0.2	1.0^b^ ± 0.1	1.1^b^ ± 0.2
Σ *n*-3	1.3^b^ ± 0.2	1.2^b^ ± 0.3	1.9^a^ ± 0.4	0.7^c^ ± 0.2	1.5^b^ ± 0.1	1.4^b^ ± 0.3

Treatments are canned bright pink salmon with 0% (B0), 1% (B1), and 2% (B2) human-grade salmon oil, and canned dark pink salmon with 0% (D0), 2% (D2), and 4% (D4) human-grade salmon oil. Different superscript letters within a row indicate significant differences (*P* < 0.05). SD, standard deviation of the mean; SAT, saturated fatty acids; MUFA, monounsaturated fatty acids; PUFA, polyunsaturated fatty acids; EPA, eicosapentaenoic acid; DHA, docosahexaenoic acid.

### Lipid hydrolysis and oxidation

**Figure 1:**
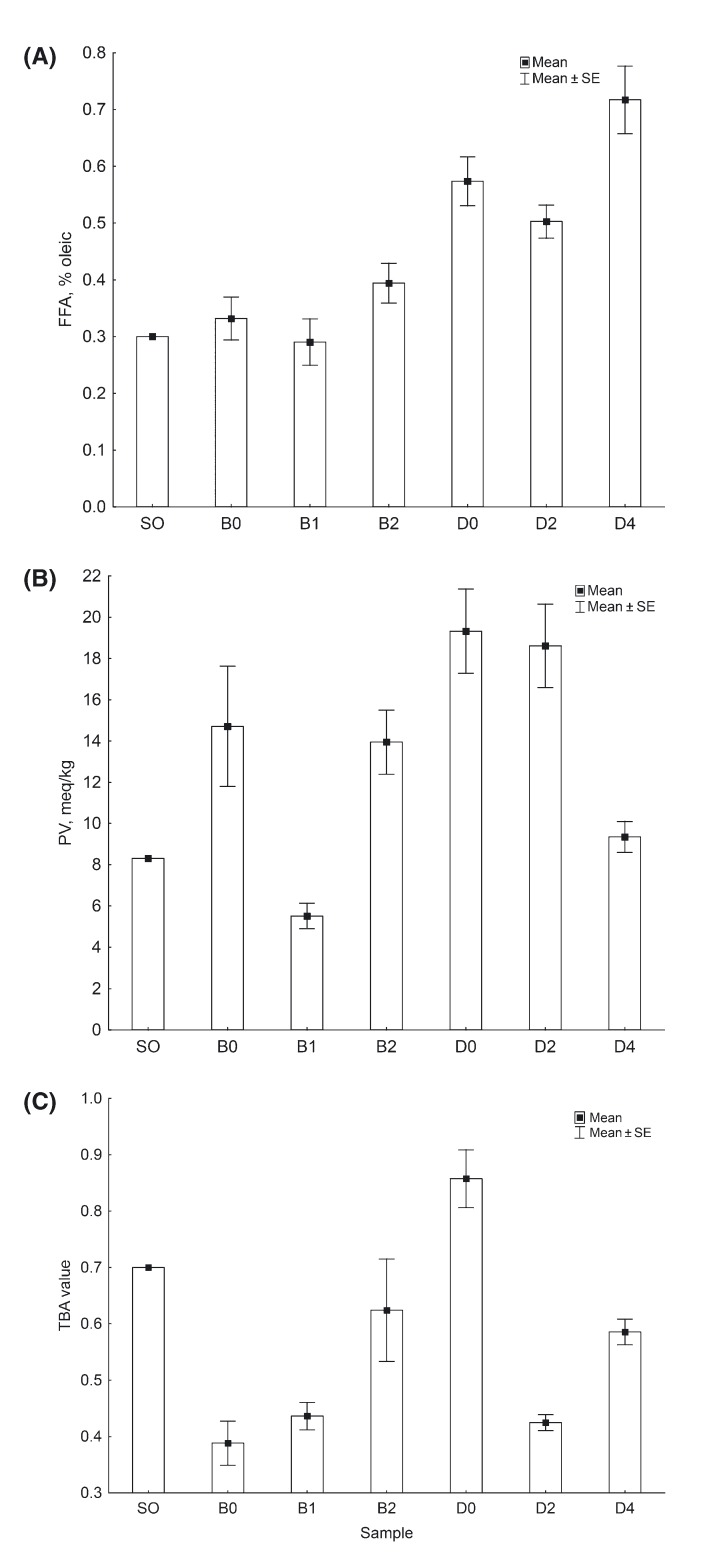
Lipid damage indices of salmon oil (SO; *n* = 1), and canned bright (B; *n *=* *12) and dark (D; *n *=* *12) Alaska pink salmon with different levels (0, 1, 2, or 4%) of salmon oil added to cans. (A) Free fatty acid values (FFA), (B) Peroxide-values (PV), (C) 2-thiobarbituric acid (TBA) values.

Lipid hydrolysis and oxidative stability analyses showed that the SO added to the canned pink salmon had not undergone significant deterioration during rendering or storage (less than 1 month from manufacturing date). The PV and FFA values for SO (Fig. 1A and B) were within the recommended quality guidelines for food-grade fish oil: 3–20 meq/kg for PV and 1–7% for FFA (Bimbo 1998).

There were significant differences (*P* < 0.05) in the extent of lipid hydrolysis among canned bright and dark Alaska pink salmon (Fig. 1A). The FFA values were similar to the range of 0.3–0.8%, previously reported for canned farmed coho salmon by Rodriguez et al. (2009). These values were lower than the 6–9% reported by Aubourg and Medina (1997) for canned tuna. Elevated FFA in the blood induces oxidative stress and promotes proinflammatory effect (Tripathy et al. 2003) leading to various organ defects, which precede type-2 diabetes (Bergman and Ader 2000) and nonalcoholic fatty liver disease (Malhi et al. 2006). Lipid oxidation was measured by evaluation of primary (PV) and secondary (TBA value) oxidation compounds (Fig. 1B and C). There were significant differences (*P* < 0.05) in PV among bright and dark canned pink salmon samples and were higher than 1.4–1.9 meq/kg determined for canned farmed coho salmon (Rodriguez et al. 2009). Although the PV values in this study were high, these were within the recommended level of 3–20 meq/kg in food-grade fish oil (Bimbo 1998). Despite significant differences observed (*P* < 0.05) in TBA values among the canned bright and dark pink salmon samples, all values were low (<1). Lipid oxidation products impart unpleasant taste and smell to the oils and exert cytotoxic and genotoxic effects (Halliwell and Chirico 1993, Esterbauer 1993). Ingestion of these compounds may cause low-density lipoprotein cytotoxicity (Morel et al. 1983), atherogenesis and atherosclerosis (Kubow 1993), and liver enlargement indicating nutrition-induced toxicity (Nwanguma et al. 1998).

Canned samples with different levels of added SO did not show patterns in lipid damage indices. Minimal oxygen availability can limit the propagation of lipid oxidation in muscle tissues (Kanner et al. 1988). As canning was done under vacuum, severe oxidation in the samples is unlikely to occur. However, low water activity (a_W_ = 0.05–0.2) of the samples due to freeze-drying may have affected the PV values, since lipid oxidation has been correlated to a_W_ (Baker et al. 2002). The a_W_ at the monolayer phase in foodstuff is considered protective from oxidation (Labuza et al. 1969), while below or above the monolayer phase the rate of oxidation increases (Labuza et al. 1969; Baker et al. 2002). In this study, the a_W_ of the canned samples were lower than the most stable condition determined for freeze-dried sockeye salmon (0.32), which was slightly above a_W_ of 0.19 for the monolayer phase (Martinez and Labuza 1968). Moreover, Aubourg and Medina (1997) reported that lipid degradation products in canned fish can either be partially destroyed by the heat treatment or interact with other constituents, such that an accurate method for their assessment is not always warranted.

### CAA test

Results in Table 4 showed that few significant differences (*P* > 0.05) were observed in the sensory attributes evaluated when canned bright pink salmon were compared against each other (B0 vs. B1 vs. B2). Sensory comparison among canned dark pink salmon (D0 vs. D2 vs. D4) showed D4 to be significantly fattier (*P* < 0.05) than D0 and D2, fishier than D0, and softer than D2. Comparison between canned bright and dark pink salmon (B0 vs. D0, B1 vs. D2, B2 vs. D4, B2 vs. D2) showed canned bright pink salmon to be significantly preferred (*P* < 0.05) in terms of color and overall taste, when compared with canned dark pink salmon. In contrast, canned dark pink salmon was found to be significantly more bitter, fattier, fishier, and softer (*P* < 0.05) than canned bright pink salmon. The sensory tests revealed that participants' favored canned salmon produced with bright fish, that is, fish that was not skin-watermarked. This finding is in line with observations regarding lower quality of late-run chum salmon muscle, which has softer texture, a gray–white color, and developed late-odor notes when canned (Huynh and Mackey 1990; Durance and Collins 1991). Overall, sensory test results indicate that panelists did not object to addition of SO to canned bright or dark pink salmon.

**Table 4:** Mean scores of sensory attributes (±SD) of the six sensory comparisons

Set	Comparison	Sample	Color	Overall taste	Bitterness	Fattiness	Fish flavor	Texture
1	B0 vs. B1 vs. B2	B0	7.5^a^ ± 3.3	9.2^a^ ± 3.0	4.6^a^ ± 3.8	5.7^a^ ± 3.3	8.2^a^ ± 3.1	7.4^a^ ± 3.1
		B1	8.2^a^ ± 3.7	9.1^a^ ± 3.1	4.1^a^ ± 3.1	5.7^a^ ± 3.5	8.0^a^ ± 3.1	7.8^a^ ± 3.6
		B2	7.1^a^ ± 3.4	8.6^a^ ± 3.3	4.2^a^ ± 3.0	6.1^a^ ± 3.3	8.2^a^ ± 3.3	7.0^a^ ± 3.4
2	D0 vs. D2 vs. D4	D0	8.1^a^ ± 3.4	8.9^a^ ± 3.1	3.9^a^ ± 3	5.6^b^ ± 3.3	8.0^b^ ± 3.1	6.8^ab^ ± 3.2
		D2	7.6^a^ ± 3.2	9.1^a^ ± 3.2	3.7^a^ ± 3.1	6.1^b^ ± 3.0	8.6^ab^ ± 3.0	7.4^a^ ± 3.5
		D4	8.5^a^ ± 3.1	8.9^a^ ± 3.2	3.8^a^ ± 3.1	7.5^a^ ± 3.8	9.1^a^ ± 2.9	6.1^b^ ± 3.3
3	B0 vs. D0	B0	8.1^a^ ± 3.2	8.6^a^ ± 3.1	3.2^b^ ± 2.4	5.2^b^ ± 2.7	7.4^b^ ± 3.1	7.7^a^ ± 3.5
		D0	6.5^b^ ± 3.1	6.6^b^ ± 3.5	4.6^a^ ± 3.5	6.3^a^ ± 3.2	8.1^a^ ± 3.5	5.3^b^ ± 3.3
4	B1 vs. D2	B1	8.3^a^ ± 3.0	9.0^a^ ± 2.9	3.4^b^ ± 2.6	5.6^b^ ± 3.2	7.6^b^ ± 3.0	7.6^a^ ± 3.4
		D2	6.3^b^ ± 3.0	6.1^b^ ± 3.6	5.1^a^ ± 3.9	6.9^a^ ± 3.4	9.0^a^ ± 3.3	4.6^b^ ± 2.9
5	B2 vs. D4	B2	8.5^a^ ± 3.1	9.9^a^ ± 2.9	3.7^b^ ± 2.9	4.8^b^ ± 2.8	8.2^b^ ± 2.9	8.3^a^ ± 3.1
		D4	5.8^b^ ± 2.9	6.3^b^ ± 3.6	5.6^a^ ± 4.0	7.2^a^ ± 3.5	9.3^a^ ± 3.3	5.1^b^ ± 3.2
6	B2 vs. D2	B2	8.8^a^ ± 3.0	10.0^a^ ± 2.9	3.9^b^ ± 3.1	5.7^b^ ± 3.3	7.6^b^ ± 3.2	8.4^a^ ± 3.1
		D2	6.5^b^ ± 3.4	7.6^b^ ± 3.4	5.2^a^ ± 4.0	7.1^a^ ± 3.5	9.0^a^ ± 2.9	5.8^b^ ± 3.1

Treatments are canned bright pink salmon with 0% (B0), 1% (B1), and 2% (B2) human-grade salmon oil, and canned dark pink salmon with 0% (D0), 2% (D2), and 4% (D4) human-grade salmon oil. Different superscript letters within a column per set of comparison indicate significant difference (*P* < 0.05); SD standard deviation of the mean.

## Conclusion

Principal variations were observed in the moisture and lipid contents of canned pink salmon products studied. Compositional analysis revealed highest lipid content in sample B2 (8.7%) and lowest lipid content in sample D0 (3.5%). Lipid content of samples B0, B1, D2, and D4 were not significantly different (*P* > 0.05) ranging from 5.7% to 6.8%. Hence, addition of SO to canned pink salmon allowed for consistent lipid content between bright and dark fish. Content of the nutritionally important components in the lipids of canned bright and dark pink salmon, EPA and DHA, were also standardized by addition of SO to product. Addition of 1 or 2% SO to canned bright pink salmon was not detrimental to the sensorial properties of the product, based on the eight sensory attributes evaluated. Although B0 and B1 were not significantly different in most of the important components, addition of 1% SO would be an assurance that nutritional claims regarding *n*-3 fatty acids, and EPA and DHA contents are consistent; thus, counterbalancing the ample natural variation found in the lipid content of both, bright and dark pink salmon. It is recommended that canned bright pink salmon be supplemented with at least 1% SO, while supplementation with 2% SO would be ideal for it guarantees a minimum quantity of 1.9 g of *n*-3 fatty acids per 100 g of product. Addition of 4% SO to canned dark pink salmon was detrimental to product texture and taste, while supplementation with 2% SO did not negatively affect sensorial properties of the product, based on the eight sensory attributes evaluated. Consequently, canned dark pink salmon should be supplemented with 2% SO for it yields consistent lipid content and assures minimum *n*-3 fatty acids content of 1.5 g per 100 g of product.
